# The impact of college students' social anxiety on human-machine trust: the mediating role of perceived intelligence and the moderating role of psychological anthropomorphism

**DOI:** 10.3389/fpsyg.2026.1786901

**Published:** 2026-05-15

**Authors:** Zihan Li, Ciming Cai, Liwei Wu, Xiao Wang

**Affiliations:** 1 School of Education and Psychology, Southwest Minzu University, Chengdu, China; 2 Key Research Institute of Humanities and Social Sciences in Southwest Minzu University, State Ethnic Affairs Commission, Chengdu, China; 3 School of Humanities and Management Sciences, Southwest Medical University, Luzhou, China

**Keywords:** Chinese college students, human-machine trust, perceived intelligence, psychological anthropomorphism, social anxiety

## Abstract

**Introduction:**

To explore the relationship between social anxiety among college students and human-machine trust, and to examine the mediating role of perceived intelligence and the moderating role of psychological anthropomorphism.

**Method:**

This study surveyed 596 Chinese college students using scales. A moderated mediation model was employed for analysis.

**Results:**

(1) Social anxiety is associated with human-machine trust, and both social anxiety and psychological anthropomorphism positively predicted perceived intelligence; (2) Perceived intelligence mediated the relationship between social anxiety and human-machine trust; (3) The moderating effect of psychological anthropomorphism on the perceived intelligence of human-machine trust is not significant; (4) Psychological anthropomorphism moderated the influence of social anxiety on perceived intelligence.

**Discussion:**

This study not only validates the applicability of CASA theory among college students with social anxiety in China but also provides insight into how these students develop their cognition of and trust in AI technology within a Chinese cultural context. It provides theoretical foundations and practical insights for developing human-computer interaction systems aligned with local sociopsychological characteristics.

## 1 Introduction

Social anxiety refers to the intense emotional reactions of worry, nervousness, or fear, along with avoidance behaviors, that individuals experience in one or more interpersonal situations (Guo, 2000). Even in non-clinical samples, symptoms of social anxiety are relatively common (Purdon et al., 2001). Research indicates that individuals' levels of social anxiety typically begin to rise gradually during childhood, peaking between the ages of 13 and 23 (Peng et al., 2004; Schmidt and Blanz, 1989). Chinese college students are currently in this high-incidence phase (Li, 2018), and the university years represent a critical period for their maturation and socialization. The complex and diverse social activities during this time can easily trigger anxiety and unease (Knapstad et al., 2021; Xu and Li, 2024). Surveys indicate that approximately 31.2% of Chinese college students experience high levels of social anxiety (Jiang et al., 2023), while 15.52% of Canadian adolescents are classified as having moderate to high risk for social anxiety (Borg et al., 2024). As a prevalent psychological trait, social anxiety not only impacts college students' daily social interaction patterns but also undermines their ability to establish and maintain healthy interpersonal relationships (O'Day and Heimberg, 2021; Yang et al., 2023). To avoid the pressures of face-to-face communication, college students with high social anxiety often prefer to rely on technological media for communication (Kong et al., 2020; Yu, 2020).

With the iterative evolution of artificial intelligence technology, generative AI chatbots, one form of AI virtual representation, have gradually integrated into college students' learning and daily life, serving multiple purposes, including information retrieval, academic assistance, and emotional support (Schei et al., 2024). For college students experiencing social anxiety, chatbots may emerge as an alternative social tool. According to the social compensation hypothesis, individuals who feel anxious or uncomfortable in real-world social interactions may turn to online socializing to seek greater control and pleasure (Davis and Kraus, 1989; Song, 2022). Research has found that chatbots are preferred by college students with social anxiety due to their safety, non-judgmental nature, anonymity, and round-the-clock availability, thereby facilitating the self-disclosure of sensitive information (Bae Brandtzæg et al., 2021; Liu et al., 2025; Meng and Dai, 2021; Skjuve et al., 2021). In the field of human-computer interaction, human-machine trust can be defined as “the attitude that an agent will help achieve an individual's goals in a situation characterized by uncertainty and vulnerability” (Lee and See, 2004), reflecting confidence in the system's safety, reliability, and predictability (Riegelsberger et al., 2005).

Although existing research has noted the preference for AI chatbots among individuals with social anxiety, studies on how this preference influences the mechanisms of human-machine trust formation remain scarce, particularly regarding its mediating and moderating effects. Based on the “Computers Are Social Actors” (CASA) theoretical framework, perceived intelligence and psychological anthropomorphism may serve as key variables explaining this process. Therefore, this study aims to examine the influence mechanism of social anxiety on human-machine trust among Chinese college students through a moderated mediation model, revealing the mediating role of perceived intelligence and the moderating effect of psychological anthropomorphism. This moderated mediation model seeks to reveal how college students with social anxiety establish trust in chatbots through perceived intelligence and whether this process varies across levels of psychological anthropomorphism. This will extend the application of CASA theory to specific populations, deepen understanding of individual differences in human-computer interaction, and provide theoretical foundations and practical insights for developing human-computer interaction systems aligned with local sociopsychological characteristics.

## 2 Review of literature and hypothesis development

### 2.1 CASA theory

The Computers Are Social Actors (CASA) theory originates from the media equation (Reeves and Nass, 1996). Its core principle is that humans treat media and computers like real people, mindlessly applying scripts for interacting with humans to interactions with social technologies (Nass and Moon, 2000; Nass et al., 1994). These computer and media agents are not entirely without limitations: they require sufficient social cues and sourcing to prevent users from perceiving them merely as information conduits. Many contemporary media agents exhibit increasingly anthropomorphic behaviors or appearances, which heightens the likelihood of eliciting the effects predicted by CASA and facilitates faster information processing (Gong and Nass, 2007). Recent researchers have proposed extensions to CASA theory. Current media agents' affordances have advanced, technical knowledge is widespread, and opportunities for human-media agent interaction have increased both in frequency and duration. Consequently, over time, users may iteratively develop “human-media” social scripts through rich interactions with media agents, replacing human-human social scripts. The antecedent of “mindlessly” then shifts to the accumulation and internalization of “human-media” social script experience (Gambino et al., 2020).

CASA is often employed to guide research situated in human-computer interaction (Gambino et al., 2020; Xue et al., 2026). Within this framework, one prominent application involves transferring the SCM social script to the AI domain to explore human cognition and attitudes toward AI (e.g., Macieira et al., 2025; McKee et al., 2023, 2024).

### 2.2 Social anxiety and human-machine trust

The Stereotype Content Model (SCM) is a classic model of interpersonal perception, suggesting that the establishment of interpersonal trust relies on perceptions of two core dimensions in the interaction partner: “competence” and “warmth” (Fiske et al., 2007; Mayer et al., 1995). According to the CASA framework, individuals with social anxiety may transfer cognitive mechanisms formed in interpersonal interactions, such as SCM, to their interactions with chatbots. Existing research demonstrates that, under the CASA framework, human cognitive mechanisms toward AI align with the dimensions of “competence” and “warmth,” thereby influencing acceptance and use of AI (Christoforakos et al., 2021; McKee et al., 2023, 2024).

Regarding human-machine trust formation, Rheu et al. (2021) reviewed empirical studies on conversational agents and found that user characteristics influence the roles of various antecedents in establishing trust. For instance, introverts place greater emphasis on an AI's instrumental intelligence rather than its social intelligence. This finding suggests that transferring the SCM to human-machine trust formation may require considering the impact of user characteristics. For individuals with social anxiety, the most critical user characteristic to consider is social anxiety itself. The core manifestations of social anxiety involve preoccupation with negative evaluation and self-doubt regarding one's social competence (Jefferies and Ungar, 2020). For college students with social anxiety, the reliability and low threat level of the interaction partner are particularly crucial (Lazarov et al., 2021; Uzun et al., 2025), potentially leading them to exhibit unique psychological mechanisms in establishing human-machine trust.

Wang et al. (2025) demonstrated through empirical studies and interviews that social anxiety indeed influences users' acceptance and trust in generative AI chatbots. Individuals with severe symptoms appreciate the empathy and non-judgmental support offered by GenAI chatbots, while those with mild symptoms prioritize technological reliability and accuracy. This reveals that individuals with social anxiety also exhibit interpersonal dynamics during AI interactions, characterized by a desire for social connection coupled with avoidance of criticism and uncertainty, consistent with the CASA framework. However, the authors did not explore the specific psychological mechanisms of trust formation from this perspective. Therefore, investigating the unique mechanisms underlying human-machine trust formation in socially anxious individuals through the CASA framework is warranted. For specific transferable cognitive mechanisms, the SCM model can serve as a reference.

### 2.3 The mediating role of perceived intelligence

Multiple studies on human-machine trust indicate that intelligence serves as a stable antecedent for trust formation (Asan et al., 2020; Glikson and Woolley, 2020). This aligns with the competence dimension of SCM within the CASA framework. The psychological representation of intelligence is perceived intelligence, which refers to users' subjective evaluations of AI capabilities (Kim et al., 2022; **?**). It reflects users' subjective perceptions of the system's competence, response accuracy, and adaptability during task execution (Pillai and Sivathanu, 2020; Qiu et al., 2020; Yu, 2019). It emphasizes evaluating whether the system “can perform tasks effectively,” without involving inferences about the system's sociality or personality traits. This constitutes a core factor in users' judgments of robotic performance. The Intelligence Scale for AI proposed by Min (2023) encompasses dimensions such as utility, security, explainability, generalization, and autonomy, providing a framework for understanding the structure of perceived intelligence.

Perceived intelligence positively influences human-machine trust (Law et al., 2022; Song and Shin, 2024). McKee et al. (2023) identified autonomy as the antecedent of competence in the SCM framework when applied to AI domains—a dimension captured by perceived intelligence. Considering the user trait of social anxiety, users with social anxiety place greater emphasis on a chatbot's non-judgmental support, technical reliability, and stability (Wang et al., 2025). Perceived intelligence manifests as high utility, security, and explainability, precisely addressing needs that socially anxious users struggle to fulfill in real-world interpersonal interactions. Therefore, it can be hypothesized that perceived intelligence stably mediates the relationship between social anxiety and human-machine trust.

### 2.4 The moderating role of psychological anthropomorphism

Anthropomorphism is one of the core concepts in human-computer interaction research (Li and Sung, 2021; Xie et al., 2023) and also serves as a crucial social cue for activating the CASA framework. Psychological anthropomorphism is defined as attributing uniquely human psychological characteristics (such as personality, cognition, empathy, etc.) to non-human entities (Shen and Zhang, 2024; Złotowski et al., 2015). The study of anthropomorphism within the CASA paradigm has suggested generally positive effects (Gambino et al., 2020). In research on human-machine trust, while intelligence consistently yields stable, positive outcomes, the impact of anthropomorphism on shaping human-machine trust is more complex. Although predominantly positive, it also carries negative implications such as the uncanny valley effect (Haugeland et al., 2022; Lee and Kim, 2025; Mori et al., 2012). The complex effects of anthropomorphism on trust primarily involve its interaction with intelligence and the influence of user characteristics (Ma et al., 2025; Przegalinska et al., 2019; Song and Shin, 2024). Glikson and Woolley (2020) reviewed prior empirical studies on AI trust, organized by AI representation form and intelligence level. They found that when virtual agents serve as the representation form, anthropomorphism may lead users to develop overly high expectations. When these expectations are mismatched with actual intelligence, trust calibration issues arise. The trust curve for AI, represented as virtual agents, declines from high to low (Glikson and Woolley, 2020). Considering social anxiety as a user characteristic, individuals with social anxiety place greater emphasis on technological reliability and stability. When trust calibration issues arise, the mismatch between intelligence and anthropomorphism may accelerate the decline of the trust curve.

McKee et al. (2023) introduced SCM into the AI domain, finding that interdependence serves as a key antecedent to warmth. And anthropomorphism serves as the cognitive prerequisite for perceptions of warmth and interdependence. Considering the unique user characteristic of social anxiety, whose psychological conflict lies in simultaneously avoiding yet desiring social interaction (Wang et al., 2025), the impact of anthropomorphism on trust may be even more significant in such contexts. Analyzing through the CASA framework, the complex influence of anthropomorphism on trust may stem from that appropriate psychological anthropomorphism satisfying social cues to generate social presence (Haugeland et al., 2022), thereby responding to users‘ desire for social interaction; However, it is also reasonable to predict that excessive anthropomorphism may trigger social cues that activate social anxiety sufferers' fear and avoidance of real-world social interactions, thereby reducing human-machine trust. However, this hypothesis currently lacks empirical research.

Anthropomorphism itself serves as a key variable in activating social cues within the CASA theory. Empirical studies indicate that Chinese college students exhibit greater acceptance of anthropomorphizing AI technologies and more pronounced psychological anthropomorphism (Folk et al., 2025; Yam et al., 2023). Therefore, psychological anthropomorphism is an indispensable factor when examining Chinese college students' chatbot usage behavior within the CASA framework. However, given the complex effects of anthropomorphism on establishing human-machine trust, as well as the significant impact of perceived intelligence and its interaction on human-machine trust, it is hypothesized that anthropomorphism acts as a moderator, influencing human-machine trust establishment through its interaction with perceived intelligence.

In recent years, new developments in the field of CASA have also provided important insights for related research, suggesting that individuals may have developed unique “human-media” social scripts (Gambino et al., 2020). For college students, frequent use of chatbots helps them clearly recognize the tools' instrumental nature, rather than unconsciously triggering social responses influenced by ingrained cognitive mechanisms. Based on this, it is hypothesized that college students with social anxiety are more likely to rely on perceived intelligence as a core mediating pathway when establishing human-machine trust with chatbots; findings by Wang et al. (2025), which indicate that individuals with mild social anxiety prioritize technical reliability and accuracy, provide indirect support for this hypothesis. Furthermore, from the perspective of social anxiety as a user trait, college students with social anxiety prefer chatbots as a form of social compensation (Skjuve et al., 2021; Song, 2022). Therefore, college students' assessment of a chatbot's competencies may focus more on its ability to fulfill this compensatory function, particularly in contexts where psychological anthropomorphism triggers significant social cues. Based on this, the present study further hypothesizes that psychological anthropomorphism and social anxiety jointly influence perceived intelligence through an interactive effect.

### 2.5 Hypothesis proposed

In summary, social anxiety, a prevalent psychological trait among college students, influences social behavior and may shape unique pathways for trust formation when interacting with chatbots. Based on CASA theory, this study constructs a mediating model of “social anxiety—perceived intelligence—human-machine trust” and introduces psychological anthropomorphism as a moderating variable to explore the differential roles of varying levels of psychological anthropomorphism in the overall mechanism of trust formation. This research not only deepens understanding of human-machine trust mechanisms among social anxiety groups within the CASA framework but also provides theoretical foundations and practical insights for developing human-computer interaction systems tailored to local sociopsychological characteristics, thereby optimizing their applications in education and psychological support.

**Figure 1:**
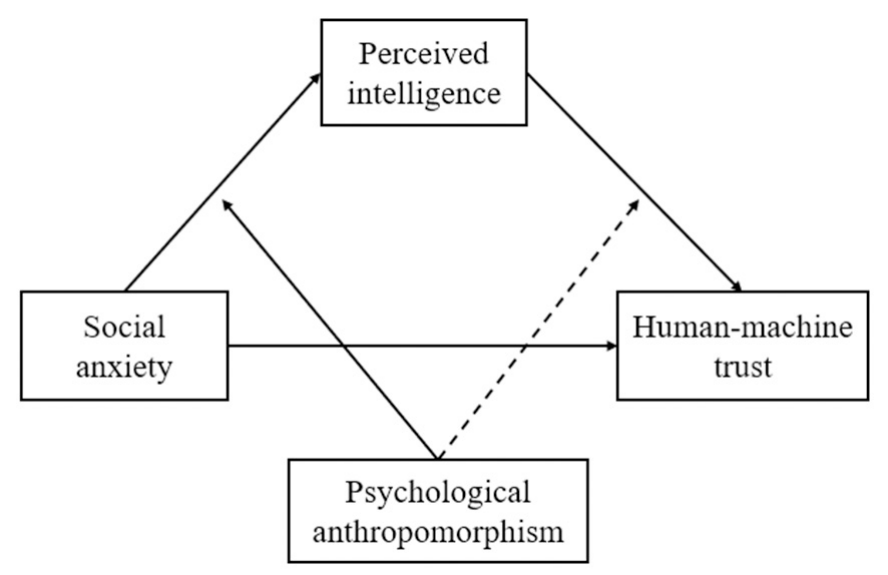
Conceptual model of a moderated mediation, in which perceived intelligence mediates the effect of social anxiety on human-machine trust. The final results indicate that this mediation is moderated by psychological anthropomorphism only in the path from social anxiety to perceived intelligence.

Therefore, we propose (Figure 1):

Hypothesis 1: Perceived intelligence mediates the relationship between social anxiety and human-machine trust.

Hypothesis 2: Psychological anthropomorphism moderates the relationship between perceived intelligence and human-machine trust, such that at high levels of psychological anthropomorphism, the positive influence of perceived intelligence on human-machine trust is weakened.

Hypothesis 3: Psychological anthropomorphism moderates the relationship between social anxiety and perceived intelligence, such that at high levels of psychological anthropomorphism, the positive influence of social anxiety on perceived intelligence is weakened.

## 3 Materials and methods

### 3.1 Participants

A questionnaire survey employing convenience sampling was conducted among 596 college students in Southwest China through online recruitment advertisements. A total of 501 valid questionnaires were finally collected after excluding invalid ones due to non-response, invariant responses, or patterned responses (DeSimone et al., 2015). Among these, 165 were male (32.93%), and 336 were female (67.07%). By major, 222 respondents (44.31%) were in natural sciences, 267 (53.29%) in humanities and social sciences, and 12 participants (2.4%) from other disciplines. The mean age of the valid sample was 21.45 ± 1.90 years (Table 1). The study strictly adhered to the principles of informed consent and anonymity. All participants read and signed an electronic informed consent form prior to completing the questionnaire, which did not include sensitive personal information. To minimize social desirability bias, the questionnaire instructions emphasized that data would be used solely for academic research and that no standard answers were provided.

**Table 1:** Sample demographic characteristics table.

Variable	Category	*n*	*%*	*M*	*SD*
**Gender**	Male	165	32.93		
	Female	336	67.07		
**Major**	Natural sciences	222	44.31		
	Humanities and social sciences	267	53.29		
	Other fields	12	2.40		
**Age**				21.45	1.90

*M* = mean; SD = standard deviation. Percentages are based on valid questionnaires, *N* = 501.

### 3.2 Measures

#### 3.2.1 Social anxiety

The Interaction Anxiety Scale (IAS), developed by Leary (1983), was revised by Li et al. (2023) and has demonstrated reliability and validity for measuring subjective social anxiety among Chinese university students (Teng et al., 2021; Yang et al., 2022). The revised IAS in the Chinese version includes 13 items scored on a 5-point Likert scale. Higher scores indicate greater subjective social anxiety levels. In this study, Cronbach's α coefficient for the IAS was 0.92, indicating good measurement quality.

#### 3.2.2 Perceived intelligence

The Intelligence Scale for AI (ISAI), developed by Min (2023), comprehensively reflects individuals‘ perceptions of other agents' intelligence. This scale comprises five dimensions: perceived utility, perceived security, perceived explainability, perceived generalization, and perceived autonomy, with 24 items scored on a 7-point Likert scale. Higher scores indicate greater perceived intelligence of AI. In this study, Cronbach's α coefficient for the ISAI was 0.92, indicating good measurement quality.

#### 3.2.3 Human-machine trust

The Human-Machine Trust Scale (HMT), developed by Malle and Ullman (2021) and translated by Min (2023), measures individuals' attitudes toward other agents as measurement objects in helping them achieve specific goals. This scale comprises two dimensions: moral trust and performance trust, with 12 items scored on a 7-point Likert scale. Higher scores indicate greater levels of human-machine trust. In this study, the Cronbach's α coefficient for the HMT was 0.93, indicating good measurement quality.

#### 3.2.4 Psychological anthropomorphism

The subscale, including two dimensions: personality anthropomorphism and empathy anthropomorphism, from the Artificial Intelligence Psychological Anthropomorphism Scale developed by Shen and Zhang (2024), comprises a total of 7 items. This scale measures the extent to which users attribute human psychological traits to artificial intelligence. Responses were recorded on a 7-point Likert scale, with higher scores indicating greater psychological anthropomorphism toward AI. In this study, the Cronbach's α coefficient for the subscale was 0.92, indicating good measurement quality.

### 3.3 Data analysis

Statistical analysis of the data was conducted using SPSS 26.0, the PROCESS 4.0 macro, AMOS 28.0, and the “Master Validity Tool” AMOS plugin. After performing common method bias tests and a confirmatory factor analysis using AMOS and its plugin, SPSS was used to analyze mediating and moderating effects.

SPSS 26.0 and the PROCESS 4.0 macro, based on ordinary least squares (OLS) regression, have a mature, extensive application foundation for handling conditional indirect effects involving mediating and moderating effects. It serves as the standard tool for examining complex causal mechanisms in the field of psychology (Hayes and Rockwood, 2017). Although the sample size (*N* = 501) in this study is sufficient to support structural equation modeling (SEM) analysis, given the focus on testing the significance of specific path coefficients and conditional indirect effects, approximate results can be obtained using SPSS and the PROCESS macro without resorting to more complex SEM methods. Therefore, the PROCESS macro, highly aligned with the testing objectives, was employed.

First, SPSS 26.0 was used to examine common method bias and perform descriptive statistics and correlation analyses among factors, including social anxiety, perceived intelligence, human-machine trust, and psychological anthropomorphism. Second, the PROCESS 4.0 macro program was used to test the mediating role of perceived intelligence in the relationship between social anxiety and human-machine trust. Finally, the moderating effect of psychological anthropomorphism on the mediating relationship was examined.

Additionally, gender and major were included as control variables, as students from different disciplinary backgrounds may exhibit variations in their familiarity with and attitudes toward technology use (Kim and Sundar, 2012).

## 4 Results

### 4.1 Common method variance analysis

Statistical tests for common method bias were conducted using the Harman single-factor test and the Unmeasured Latent Factors (ULMCF) method. Results indicate that 9 factors with eigenvalues exceeding 1 collectively explained 66.84% of the variance. The unrotated first factor accounted for 32.64% of variance, falling below the 40% critical threshold. These findings indicate that the problem of common method variance in this study was not serious. Moreover, the results of confirmatory factor analysis (Table 3) indicate that the fit of the four-factor model (χ^2^*/df* = 2.54, TLI = 0.87, CFI = 0.88, RMSEA = 0.056) is significantly superior to that of the one-factor model (Δχ^2^ = 10,647.53, Δ*df* = 15, *p* < 0.05). Furthermore, the factor model derived using the ULCMF method did not show significant superiority over the four-factor model (ΔTLI = 0.02 < 0.1, ΔCFI = 0.02 < 0.1, ΔRMSEA = 0.005 < 0.05). Taken together, these results indicate that the common method variance of the measures did not reach a severe level and had a minimal impact on the reliability of the study findings (Baron and Kenny, 1986; Harrison et al., 1996; Podsakoff et al., 2003; Wen et al., 2018).

### 4.2 Confirmatory factor analysis

This study used composite reliability (CR) and average value extracted (AVE) from exploratory factor analysis to assess the scales' validity (Table 2). As shown, only two factors in this study had AVEs greater than 0.5, but all had CRs greater than 0.7. Overall, the composite reliability and convergent validity of the variables in this study are considered acceptable (Nguyen, 2020; Verhoef et al., 2002).

**Table 2:** AVE values and composite reliability for the variables.

Variable	CR	AVE
1 Social anxiety	0.92	0.47
2 Perceived intelligence	0.80	0.45
3 Human-machine trust	0.81	0.68
4 Psychological anthropomorphism	0.88	0.79

This study also employed confirmatory factor analysis to examine the discriminant validity among the latent variables in the model, including social anxiety, perceived intelligence, human-machine trust, and psychological anthropomorphism (Table 3). As shown in the table, the four-factor model fits well, with fit indices significantly superior to those of the other four competing models, indicating that the four latent variables are independent of one another. Furthermore, HTMT values were calculated using the Master Validity Tool plugin, and the HTMT values between all variables were less than 0.9. Taken together, these results indicate that the variables in this study exhibit acceptable discriminant validity (Henseler et al., 2015; Hsu et al., 2015; Hu et al., 2023).

**Table 3:** The result of confirmatory factor analysis.

Model	*χ^2^/df*	TLI	CFI	RMSEA	SRMR
One-factor model (A+B+C+D)	7.175	0.497	0.515	0.111	0.123
Two-factor model (A, B+C+D)	5.296	0.650	0.663	0.093	0.088
Three-factor model a) (A, B+C, D)	5.118	0.664	0.677	0.091	0.088
Three-factor model b) (A, B, C+D)	4.999	0.674	0.687	0.089	0.087
Four-factor model (A, B, C, D)	2.547	0.874	0.880	0.056	0.072

**A** represents social anxiety, **B** represents psychological anthropomorphism, **C** represents perceived intelligence, and **D** represents human-machine trust.

### 4.3 Descriptive statistics and correlation analysis

The correlation analysis results indicate that social anxiety shows significant positive correlations with perceived intelligence, human-machine trust, and psychological anthropomorphism. Perceived intelligence shows significant positive correlations with human-machine trust and psychological anthropomorphism, while human-machine trust correlates positively with psychological anthropomorphism (Table 4). These findings suggest that social anxiety, perceived intelligence, human-machine trust, and psychological anthropomorphism are not isolated phenomena but are instead closely interconnected and mutually influential.

**Table 4:** Descriptive statistics and correlation coefficients for the variables.

Variable	*M*	*SD*	1	2	3	4
1 Social anxiety	39.30	10.62	1			
2 Perceived intelligence	113.09	20.54	0.19^**^	1		
3 Human-machine trust	56.44	12.73	0.22^**^	0.80^**^	1	
4 Psychological anthropomorphism	31.10	9.08	0.23^**^	0.76^**^	0.79^**^	1

M = mean; SD = standard deviation. ^**^p < 0.01. *N* = 501.

### 4.4 Analysis of the moderated mediation model

First, Model 4 of the PROCESS 4.0 macro was applied to examine the mediating role of perceived intelligence in the relationship between social anxiety and human-machine trust. Social anxiety had a significant total effect on human-machine trust [β = 0.25, *p* < 0.001, 95% CI (0.15, 0.35)]. After including perceived intelligence as a mediator, social anxiety significantly predicted perceived intelligence [β = 0.36, *p* < 0.001, 95% CI (0.19, 0.52)], indicating that higher levels of social anxiety were associated with higher perceived intelligence. Perceived intelligence significantly predicted human-machine trust [β = 0.49, *p* < 0.001, 95% CI (0.45, 0.52)], indicating that higher perceived intelligence was associated with greater human-machine trust. Social anxiety still positively predicted human-machine trust [β = 0.08, *p* < 0.05, 95% CI (0.01, 0.14)] (Table 5). The bias-corrected Bootstrap test indicated that the mediation effect value of perceived intelligence was 0.17, with a Bootstrap (5,000 samples) 95% confidence interval [0.08, 0.26], and a relative mediation effect of 69.04% (Table 6). This indicates that perceived intelligence partially mediates the relationship between social anxiety and human-machine trust, validating Hypothesis 1. Specifically, college students with higher social anxiety are more likely to perceive chatbots as more intelligent, and this perception enhances their trust in chatbots during use.

**Table 5:** Test of the mediating effect of perceived intelligence.

Antecedent	Perceived intelligence			Human-machine trust		
	β	*t*	95%CI	β	*t*	**95%CI**
Social anxiety	0.36	4.19^***^	[0.19,0.52]	0.08	2.36^*^	[0.01,0.14]
Perceived intelligence				0.49	28.53^***^	[0.45,0.52]
Gender	0.98	0.50	[−2.86,4.82]	−0.02	−0.03	[−1.48,1.43]
Major	−5.67	−3.41^***^	[−8.94, −2.40]	−0.77	−1.20	[−2.02,0.49]
*R^2^*	0.06			0.65		
*F*	10.78^***^			228.35^***^		

^*^p < 0.05, ^***^p < 0.001.

**Table 6:** Total, direct, and indirect effects of perceived intelligence.

	Effect	*BootSE*	*BootLLCI*	*BootULCI*	Ratio
Total effect	0.25	0.05	0.00	0.15	
Direct effect	0.08	0.03	0.02	0.01	30.96%
Indirect effect	0.17	0.05	0.08	0.26	69.04%

Model 59 of the PROCESS 4.0 macro was applied to analyze the moderating effect of psychological anthropomorphism on the mediation model. Psychological anthropomorphism did not significantly moderate the relationship between perceived intelligence and human-machine trust in the mediation model [β = 0.00, *p* > 0.05, 95% CI (-0.003, 0.003)], thus failing to validate Hypothesis 2; psychological anthropomorphism does not simultaneously moderate both parts of the mediation model, and the direct effect of the interaction between perceived intelligence and psychological anthropomorphism on human-machine trust was not significant. However, psychological anthropomorphism was found to moderate the relationship between social anxiety and perceived intelligence in the mediation model, suggesting that Hypothesis 3 may hold. This indicates that psychological anthropomorphism does not diminish college students' trust in robots because their perceived intelligence falls short of expectations. However, it may influence their perception of the chatbot's intelligence.

To focus on testing Hypothesis 3, model 7 of the PROCESS 4.0 macro was applied to analyze the moderating effect of psychological anthropomorphism on the mediation model. The interaction term between social anxiety and psychological anthropomorphism significantly moderates the effect of perceived intelligence [β = −0.02, *p* < 0.01, 95% CI (−0.03, −0.01)], suggesting that the relationship of perceived intelligence and social anxiety is moderated by levels of psychological anthropomorphism. Social anxiety significantly influenced human-machine trust [β = 0.08, *p* < 0.05, 95% CI (0.01, 0.14)], while perceived intelligence also significantly affected human-machine trust [β = 0.49, *p* < 0.001, 95% CI (0.45, 0.52)] (Table 7). This indicates that psychological anthropomorphism exerts a negative moderating effect on the first half of the model. When psychological anthropomorphism is low, college students with higher social anxiety are more likely to perceive the chatbot as intelligent. However, when college students exhibit higher levels of psychological anthropomorphism toward chatbots, college students with higher social anxiety are less likely to perceive the chatbot as more intelligent.

**Table 7:** The moderated mediating model.

Antecedent	Perceived intelligence			Human-machine trust		
	β	*t*	95%CI	β	*t*	95%CI
Social anxiety	0.09	1.54	[−0.03,0.21]	0.08	2.36^*^	[0.01,0.14]
psychological anthropomorphism	1.65	23.85^***^	[1.52,1.79]			
Social anxiety × Psychological anthropomorphism	−0.02	−3.04^**^	[−0.03,-0.01]			
Perceived intelligence				0.49	28.53^***^	[0.45,0.52]
Gender	−1.40	−1.07	[−3.96,1.16]	−0.02	−0.03	[−1.48,1.43]
Major	−2.07	−1.86	[−4.26,0.12]	−0.77	−1.20	[−2.02,0.49]
*R^2^*	0.59			0.65		
*F*	140.56^***^			228.35^***^		

^*^p < 0.05, ^**^p < 0.01, ^***^p < 0.001.

**Figure 2:**
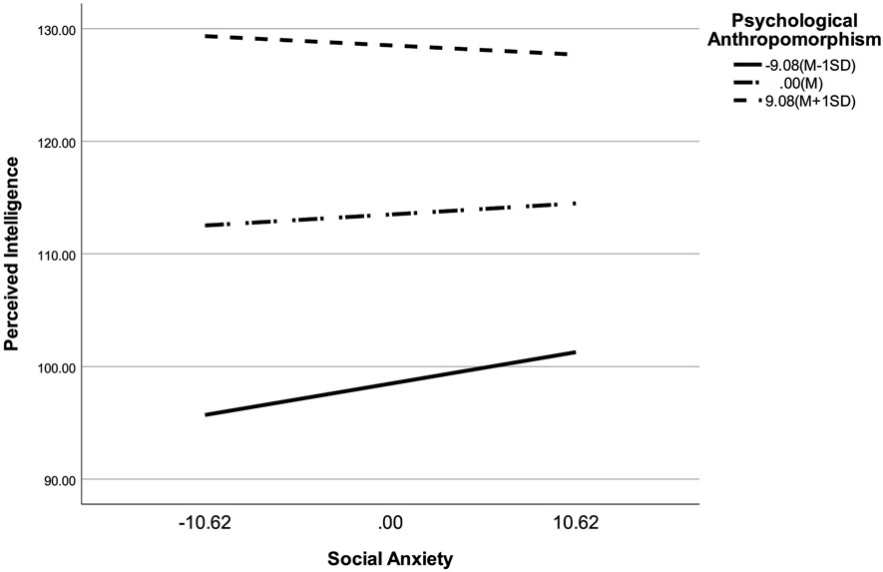
The figure shows the interaction between social anxiety and psychological anthropomorphism on perceived intelligence, specifically the simple slopes of social anxiety predicting perceived intelligence at low (−1 SD), mean, and high (+1 SD) levels of psychological anthropomorphism.

A further simple effects analysis examined the predictive effect of social anxiety on perceived intelligence at psychological anthropomorphism levels one standard deviation below the mean, at the mean, and one standard deviation above the mean. The predictive role of social anxiety on perceived intelligence was strongest at low levels of psychological anthropomorphism (β = 0.26, *t* = 2.85, *p* < 0.01), non-significant at average levels (β = 0.09, *t* = 1.54, *p* = 0.12), and non-significant at high levels (β = −0.08, *t* = −1.09, *p* = 0.27). Examining the indirect effect of social anxiety on human-machine trust via perceived intelligence at different levels of psychological anthropomorphism, the effect sizes were 0.13, 0.05, and −0.04 for psychological anthropomorphism above average, average, and below average, respectively (Table 8). This indicates that as psychological anthropomorphism tendencies increase, the predictive power of social anxiety on perceived intelligence diminishes (Figure 2).

**Table 8:** The mediating effect at different levels of psychological anthropomorphism.

	Psychological anthropomorphism	Mediating effect	*BootSE*	*BootULCI*	*BootULCI*
The moderated mediation effect	*M+1SD*	−0.04	0.04	−0.11	0.03
	*M*	0.05	0.03	−0.02	0.11
	*M-1SD*	0.13	0.05	0.02	0.23

M = mean; SD = standard deviation.

## 5 Discussion

This study, grounded in the CASA theoretical framework, systematically examined the association between social anxiety on human-machine trust by constructing a moderated mediation model using a sample of 501 college students from Southwest China. It explored the applicability of CASA theory among groups with characteristics similar to those of college students in Southwest China and showed patterns in how cognitive pathways and trust-building processes toward AI technology are reflected among the Chinese college students.

Research indicates that perceived intelligence reflects college students' instrumental assessment of a robot's task efficacy, representing a competence pathway through which individuals with social anxiety relate to initial trust in chatbots. Concurrently, psychological anthropomorphism reflects the degree to which college students attribute social characteristics to the system, representing a relational pathway. By providing social presence (Haugeland et al., 2022; Konya-Baumbach et al., 2023) that satisfies emotional needs, it is linked to a weaker role of perceived intelligence. These findings are consistent with the “competence-warmth” dual-dimensional trust model (Fiske et al., 2007), suggesting that both pathways may interact synergistically rather than operating as mutually exclusive alternatives in establishing human-machine trust among individuals with social anxiety. Furthermore, these results provide support for the core assumption of CASA theory, namely that interpersonal trust mechanisms may extend to human-computer interaction contexts.

The incomplete validation of Hypothesis 2 may be related to sample characteristics. First, this study primarily involved college students from Southwest China with relatively homogeneous cultural and educational backgrounds, resulting in lower variance in trust judgments and reduced interaction effect significance. Secondly, the era and cultural level of the sample may also play a role. The sample consists of university students in 2025, who can be considered the digital natives of the digital era. Moreover, in the era of rapid iteration in AI development, university students have greater access to technical knowledge and more opportunities to use it than non-technical groups. They have ample access and opportunities to various AI technologies and engage in long-term interactions. This satisfies the conditions for establishing the “human-media” social script in the new CASA extension (Gambino et al., 2020). This suggests that college students may have developed self-consistent “human-media” scripts rather than merely initial social scripts. Specifically, in long-term interactions, while students may attribute some human-like traits to chatbots, they recognize their fundamental tool-like nature. Consequently, they tend to prioritize technical accuracy and stability, primarily building trust through competence pathways. This may mitigate the sense of loss stemming from excessive anthropomorphism, potentially influencing perceptions of intelligence without undermining trust formation. Although technological advancement is a common feature of the digital native era, China's vast territory calls for caution in applying this explanation. Given the economic and cultural disparities among university students across different regions, this interpretation is prudently limited to student groups with characteristics similar to those of the sample.

## 6 Limitations and future work

### 6.1 Future-oriented suggestions

#### 6.1.1 Cultural perspective

The triggering of CASA is also influenced by individual cultural backgrounds (Gambino et al., 2020; Nass and Moon, 2000). Unfortunately, this study did not directly examine the influence of cultural factors such as “Mianzi” and “Renqing” using quantitative methods; therefore, the following exploration is based solely on explanatory speculation, with the hope that it may inspire future research.

The internalization of the “human-media” script can also be reasonably explained within the context of China's specific cultural environment, providing a complementary perspective for understanding the data performance in this sample. On the one hand, within China's collectivist culture, which highly values interpersonal harmony and “Mianzi” (Yan et al., 2007; Zhang J. C., 2019; Zhang X. S., 2019), individuals with social anxiety often fear negative evaluations from others more intensely. Consequently, they may exhibit stronger trust tendencies toward chatbots that do not involve social judgment (Ali et al., 2024; Fan and Chang, 2015; **?**). On the other hand, prior research indicates that Chinese users particularly value technological practicality and reliability (Tian et al., 2023; Wang et al., 2022; Wilson et al., 2021). This orientation may have facilitated the internalization of a new “human-media” social script that emphasizes technological capability during high-frequency use.

The moderating effect of psychological anthropomorphism can be speculated and examined from the perspective of China's distinctive “Guanxi orientation”: High psychological anthropomorphism elicits greater social expectations toward chatbots, and users with social anxiety, lacking confidence in their social skills, may attempt to use them in specific task contexts such as relationship maintenance. If their responses fail to align with the “Renqing” in Chinese interpersonal interactions, their perceived intelligence is more likely to be questioned (Ren et al., 2021; Cao et al., 2023). Future research could incorporate cultural variables (e.g., individualism-collectivism, Mianzi consciousness, or Guanxi tendency) and test them as moderating or mediating variables in model testing. This would provide a more direct insight into the specific pathways through which human-machine trust is formed in different situational tasks under the CASA framework when influenced by cultural factors, as well as the concrete content of potential “human-media” scripts.

#### 6.1.2 Design recommendations

Based on the latest research findings in this field and the conclusions of this study, we offer the following forward-looking practical recommendations, which we hope will contribute to the development of localized chatbots and promote their healthy use among Chinese college students.

This study suggests that the development of localized chatbots in China should strike a balance between intelligence and anthropomorphism. While ensuring core intelligence, anthropomorphic cues should be designed with caution to avoid excessive human-like traits. Cultural translation must be prioritized to construct interaction logic aligned with Chinese social contexts (such as “Renqing”), achieving “empathy” without inducing pressure.

For users with social anxiety, encouraging phrasing should be integrated into intelligent, non-judgmental interaction frameworks. This approach safeguards sensitive factors like “Mianzi” and enhances the chatbot's function as a “social avoidance tool.” Algorithm training must incorporate locally prevalent high-anxiety traits to Chinese society (e.g., group pressure, comparative psychology, loss of Mianzi) to provide targeted support.

Furthermore, systematically cultivate AI literacy among college students, guiding them to view chatbots as adaptive tools for self-improvement rather than overly anthropomorphized entities, thereby internalizing reasonable “human-media” scripts. This approach aligns with the pragmatic philosophy of “making the best use of everything,” thereby establishing reasonable human-machine trust and providing guidance for human-computer interaction systems with localized psychological adaptability.

### 6.2 Limitations

Although this study provides preliminary empirical support for the relationship between social anxiety and human-machine trust, several limitations exist. Future research may explore the following areas in greater depth.

First, the cross-sectional design of this study limits causal inferences about variables. While this approach provides robust estimates of path coefficients, it fails to assess the overall fit of the measurement model simultaneously. Future work could incorporate longitudinal tracking or experimental interventions to dynamically examine the psychological mechanisms underlying the formation and evolution of human-machine trust in individuals with social anxiety, thereby further ruling out competing explanations. Furthermore, future studies should consider employing structural equation modeling (SEM) to conduct overall goodness-of-fit tests of the theoretical model developed in this study, while controlling for measurement error. This approach would enable more precise causal analysis and the examination of complex relationships among latent variables.

Second, the study primarily relies on self-report scales. While this method facilitates control over variables, it may be subject to social desirability bias or merely reflect participants' abstract conceptions of AI, resulting in limited ecological validity. In this study, while the increases in CFI and FLI resulting from the use of the ULCMF factor model did not reach 0.1, they did exceed 0.01. Therefore, it must acknowledge that although the common method bias did not reach a level significant enough to affect the analysis results seriously, it does indeed exist. Furthermore, this study focuses on general interactions without specifying usage contexts; different task objectives may trigger distinct trust-building pathways. Future research could adopt more ecologically valid paradigms in which participants interact with functional prototype chatbots during real-world tasks (e.g., information retrieval, emotional sharing) across varied scenarios. Immediately post-interaction, measures of trust, perceived intelligence, and related behavioral indicators should be collected to obtain observational data closer to real behaviors.

Furthermore, the Intelligence Scale for AI employed in this study primarily reflects users' “instrumental capability judgments” regarding a system's reliability and safety in task execution. Existing scales struggle to clearly distinguish between “perceived instrumental intelligence” and “perceived social intelligence” (Huang and Rust, 2018). Two distinct psychological representations. Such scales cannot directly determine whether college students with social anxiety base their human-machine trust formation on “performance evaluation” or “mental projection,” which represent different trust-building mechanisms and may give rise to more complex CASA scripts. Future research should further distinguish these cognitive pathways at the scale development or experimental manipulation level to more clearly reveal the independent roles of different types of perception within trust mechanisms.

Finally, the sample was primarily recruited through online platforms and predominantly sourced from universities in the southwestern region, potentially introducing tech-savvy bias. Given that college students from different regions and institutions may vary significantly in terms of socio-economic background, cultural beliefs, and exposure to technology. Differences in the social pressures students face across economic levels may also lead to variations in social anxiety levels. Therefore, this study cautiously restricts its conclusions to “groups with characteristics similar to those of college students in Southwest China”. Future research could enhance model representativeness and robustness through multi-source, multi-stage stratified sampling and cross-cultural comparisons, encompassing diverse regions and types of institutions, particularly by incorporating samples from cultural contexts outside China.

## Data Availability

The raw data supporting the conclusions of this article will be made available by the authors, without undue reservation.
